# Cell Based Autologous Immune Enhancement Therapy (AIET) after Radiotherapy in a Locally Advanced Carcinoma of the Cervix 

**DOI:** 10.1155/2013/903094

**Published:** 2013-04-07

**Authors:** Sumana Premkumar, Vidyasagar Devaprasad Dedeepiya, Hiroshi Terunuma, Rajappa Senthilkumar, Thangavelu Srinivasan, Helen C. Reena, Senthilkumar Preethy, Samuel J. K. Abraham

**Affiliations:** ^1^Chennai Meenakshi Multispeciality Hospital Limited, New No. 72, Old No. 148, Luz Church Road, Mylapore, Chennai 600 004, India; ^2^Dr. Kamakshi Memorial Hospital, 1 Radial Road, Pallikaranai, Chennai 600100, India; ^3^Nichi-In Centre for Regenerative Medicine (NCRM), PB 1262, Nungambakkam, Chennai 600034, India; ^4^Biotherapy Institute of Japan, No. 8, No. 4 Edagawa 2-chome, Koto-ku, Tokyo 135-0051, Japan; ^5^Hope Foundation (Trust), B6, 13, Zakariah Colony III Street, Choolaimedu, Chennai 600094, India; ^6^School of Medicine, Yamanashi University, 1110 Shimokato, Chuo, Yamanashi 409-3898, Japan; ^7^The Mary-Yoshio Translational Hexagon (MYTH), Nichi-In Centre for Regenerative Medicine (NCRM), PB 1262, Nungambakkam, Chennai, Tamil Nadu 600034, India

## Abstract

Radiotherapy is the primary form of treatment in patients with locally advanced cervical carcinoma. However for residual disease in the form of the persistent lymph nodes, surgery or chemotherapy is recommended. As surgery is not acceptable by every patient and chemotherapy has associated side effects, we hereby report the positive outcome of *in vitro* expanded natural killer cell and activated T lymphocyte based autologous immune enhancement therapy (AIET) for the residual lymphadenopathy in a patient with locally advanced cervical cancer after radiation. After six transfusions of AIET, there was complete resolution of residual lymph nodes and there was no evidence of local lesion. The patient also reported improvement in quality of life. As AIET has been reported as the least toxic among the available therapies for cancer, combining AIET with conventional forms of therapy in similar patients might not only improve the outcome but may also help the patients achieve a good quality of life.

## 1. Introduction

Cancer Statistics in 2008 place cervical cancer as the third most common cancer in women with more than 85% of the global burden occurring in developing countries [1]. There are 529,512 new cases of cervical cancer diagnosed per year globally and 86% of all deaths caused by cervical cancer occur in developing countries [2]. Currently radiotherapy remains the primary treatment of choice for advanced cervical carcinoma patients [2]. Autologous cell based immunotherapy represents one of the potential treatment options for several types of cancers, with randomized clinical trials published on its safety and efficacy [3–5]. We have earlier reported the positive outcome following autologous immune enhancement therapy (AIET) in pancreatic cancer [6] and ovarian cancer [7] patients. Herein we report the outcome of AIET following radiotherapy in a Stage IV-A cervical cancer patient of Indian origin.

## 2. Case Presentation

**Figure 1:**
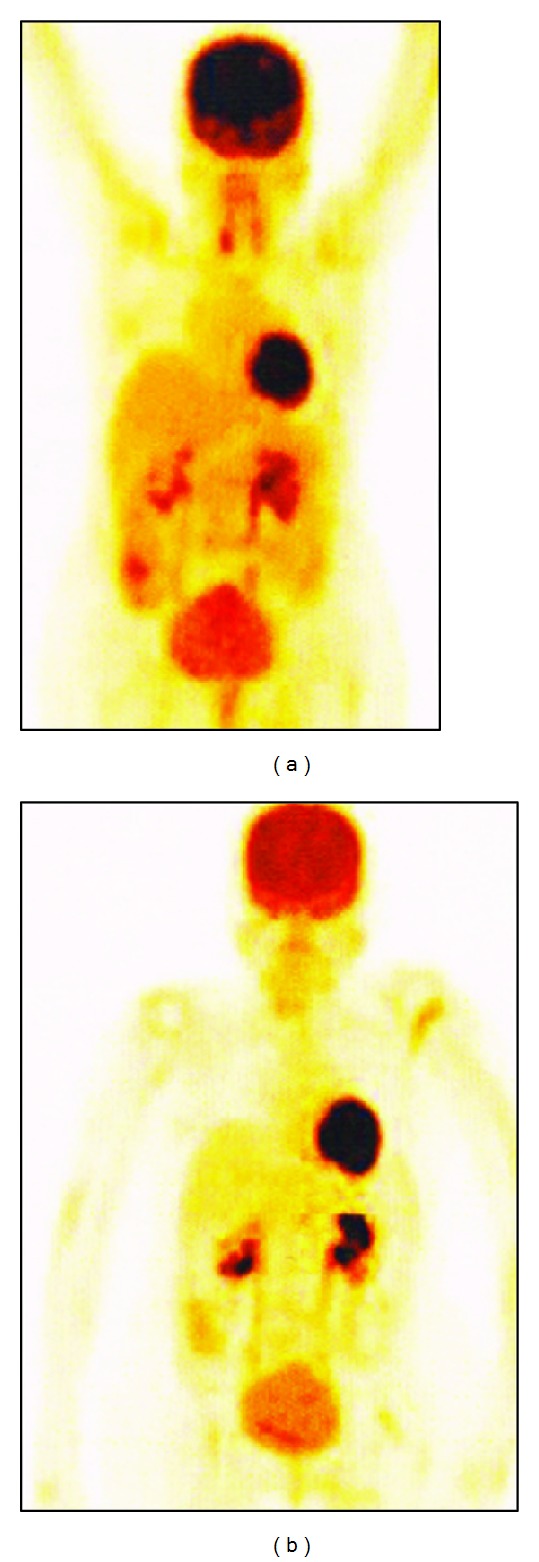
PET-CT images (a) before and (b) after immunotherapy showing the resolution of the lymph node lesions after immunotherapy.

A 58-year-old female patient presented with pain in abdomen and urinary incontinence with hematuria in April 2012 for which she was evaluated. Past medical history revealed that she had bilateral hydronephrosis for which she had undergone stenting. She also had Parkinson's disease and rheumatoid arthritis for which she was under regular medical management. A CT scan was taken which revealed a large ill-defined irregular mass in the uterine cervix with bilateral parametrial infiltration. The mass measured 5 × 4 cm at its widest point, infiltrating the lower uterine myometrium, extending anteriorly infiltrating the posterior wall and trigone of the bladder involving both vesicoureteric junctions. Posteriorly, the mass had infiltrated the anterior wall of rectum. Few enlarged internal ileac nodes were present bilaterally. Biopsy revealed the mass to be squamous cell carcinoma. She also had bilateral L5 spondylosis with grade II anterolisthesis of L5 over S1. She was treated with external radiation to the pelvis with a dose of 6000 cGy in May 2012. After radiotherapy, the PET-CT in June 2012 (Figure 1(a)) showed residual cervical thickening and residual retroperitoneal lymphadenopathy with increased F18-FDG uptake in the aortocaval and retrocaval lymphnodes with the largest measuring 1.5 × 1.4 cm. Degenerative changes were also noted in the spine. For the residual lymphadenopathy, considering the factors like invasive nature of the surgery and the comorbid conditions of the patient along with fragile nature of her physique making chemotherapy, a high-risk one, the treating physicians recommended autologous natural killer (NK) cell and activated T lymphocyte based immunotherapy termed as autologous immune enhancement therapy (AIET). The first AIET cell transfusion was done in July 2012 and the second and third cell transfusions in August 2012. For each transfusion, approximately 70 mL of peripheral blood was withdrawn and the isolation, *in vitro* expansion cum activation of the NK cells and T lymphocytes were done as reported earlier [8]. After the three transfusions, an MRI was taken in September 2012 which showed regression of the aortocaval node to 1.4 × 1 cm (earlier 1.5 × 1.4 cm) and the retrocaval node measured 0.9 × 0.5 cm. The fourth and fifth AIET transfusions were done in October 2012 and the sixth transfusions in November 2012. An average of 282.5 million NK cells and 478.5 million activated T lymphocytes were transfused in these six AIET cycles. A PET-CT scan in November 2012 after the completion of all the six cycles of AIET showed complete resolution of the retroperitoneal lymphnodes with no evidence of local lesion in the cervix (Figure 1(b)). The PET-CT also showed sclerotic lesion in the C7 vertebral body. However biopsy of the lesion was not done and the patient is still under followup. In addition to the above changes, the patient also reported improvement in quality of life after the AIET. There were no adverse reactions reported after AIET.

## 3. Discussion

Conventional therapies for cervical cancer include radical surgery, radiotherapy, and chemotherapy. Radiotherapy continues to be the preferred form of therapy for advanced cervical cancer [2]. Earlier studies report a median progression free survival of nearly 10 months and overall survival of 21 months in Stage IV-A cervical cancer patients with radiotherapy alone [9]. It has been reported that pelvic lymph node involvement persists in about 16% of the cervical cancer patients after chemoradiation [10]. As lymph node involvement is an important prognostic feature in cervical cancer [11], treatment aimed at removing the lymph nodes involved or controlling the lymph nodal metastasis becomes essential. Usually surgery or chemotherapy is the treatment of choice in such cases. These therapies are associated with potential toxic/side effects. As the patient also had comorbid conditions apart from the above issues of concern, AIET was recommended. After six cycles of AIET, there is a complete resolution of the disease in the lymph nodes involved. The residual thickening observed immediately after the radiation also resolved and there were no adverse effects during the transfusions. As this cell based AIET has been safe and the least toxic of cancer therapies similar to what is reported earlier [12], combining immunotherapy with conventional therapies is suggested for similar cases of locally advanced cervical cancer for early achievement of positive treatment outcome. Further followup of the patient will reveal the significance of immunotherapy in terms of disease free interval and long-term survival benefits.

## 4. Conclusion

NK cell and activated T lymphocytes based AIET following radiotherapy in our experience with this case did not have any adverse reaction and has resulted in resolution of the residual metastatic lymphadenopathy in a locally advanced cervical carcinoma, and the patient's general condition is continuing to be optimal throughout. Hence combining AIET with radiotherapy or other conventional therapies could be worth considering for similar patients in the future.
